# Beyond the Physical: Understanding the Emotional, Psychological, and Social Factors Affecting Women’s Health Today

**DOI:** 10.7759/cureus.81874

**Published:** 2025-04-08

**Authors:** Abeera Jamil

**Affiliations:** 1 Obstetrics and Gynaecology, Lyell McEwin Hospital, Adelaide, AUS

**Keywords:** health literacy, mental health outcomes, psychological well-being, social determinants of health, women’s health

## Abstract

We aimed to assess the emotional, psychological, and social factors affecting women’s health today, focusing on their impact on overall well-being and the extent to which these nonphysical determinants influence health outcomes. This review will synthesize findings from in-depth interviews, focus group discussions, and narrative analysis to provide insight into women’s experiences and perceptions of their health, including emotional, psychological, and social challenges in Saudi Arabia. This qualitative review will utilize a rigorous, iterative search across PubMed/MEDLINE, Scopus, PsycINFO, and Web of Science from 2000 to 2023, supplemented by grey literature searches via Google Scholar and relevant organizational websites, to identify studies employing in-depth interviews, focus groups, or narrative analysis, focusing on qualitative studies examining women's health across various ages, backgrounds, and socioeconomic statuses. Two independent reviewers will screen studies, resolve discrepancies via discussion or a third reviewer, and critically appraise study quality using the CASP Qualitative Checklist to ensure the credibility and trustworthiness of the synthesized findings. Data extraction will be structured to capture rich, descriptive data related to women’s experiences and perceptions, and NVivo software will support thematic synthesis, with a reflective journal maintained throughout the process to ensure transparency and acknowledge potential researcher biases. A narrative synthesis will be included to show the relationships between the developed themes, and how they relate to the women participating in the various studies. The qualitative review revealed that women’s health is profoundly influenced by a complex interplay of emotional, psychological, and social factors. The analysis identified six key themes: (1) societal expectations and gender roles, (2) access to healthcare and resources, (3) emotional well-being and mental health, (4) social support networks and relationships, (5) cultural and environmental factors, and (6) empowerment and autonomy. The findings highlighted the significance of addressing these factors to improve women’s health outcomes, emphasizing the need for holistic and culturally sensitive approaches to healthcare that prioritize women’s emotional, psychological, and social well-being. The findings highlight the need for integrated healthcare strategies addressing these dimensions, promoting mental health awareness, and providing support systems to enhance overall well-being. Better women’s health outcome requires education and policy-level interventions in diverse settings.

## Introduction and background

Introduction

The objective of this study is to study the emotional, psychological, and social factors that affect women’s health and assess nonphysical determinants that shape health among women. Although women’s health receives more and more attention globally, the current body of work continues to center on the physical health of women, and the neglect of important but often ignored features and unused aspects of emotional well-being, psychological resilience, and social support systems. Through investigating these dimensions, this study aims to provide a holistic understanding of women’s health and see what kind of suggestions can be made for the holistic approach to healthcare policies and interventions.

The term women’s health refers to the concept of multidimensional factors of a woman’s physical, emotional, psychological, and social well-being. Recent data showed a concern about women’s mental health. In the Saudi National Mental Health Survey, the percentage of those who exhibited moderate to severe psychological distress, including anxiety and depression was 38.1% [1]. The importance of dealing with mental health issues in the female population is highlighted by these findings.

There is a significant effect of the social determinants on health outcomes as well. Women’s health is significantly impacted by socioeconomic status (SES), education level, and employment opportunities. According to Saudi Family Health Survey data, the prevalence of NCDs was higher for women and for those with lower educational attainment in particular.

Women with education below the primary school level experienced a 1.67 times higher occurrence of non-communicable diseases (NCDs) (odds ratio (OR): 1.67, 95% CI: 1.38-2.01) compared to those with higher education [2]. This significant disparity likely reflects several interconnected factors. Limited health literacy among women with lower education may hinder their understanding of disease prevention, healthy lifestyles, and early warning signs of NCDs. Furthermore, reduced access to preventive healthcare services, often correlated with lower SES, can limit opportunities for early detection and management of risk factors.

This finding underscores the well-established association between lower SES and poorer health outcomes for women. Lower educational attainment is frequently linked to reduced employment opportunities, lower income, and limited access to resources crucial for maintaining good health, such as nutritious food and safe living environments. Consequently, women in lower socioeconomic strata may face increased exposure to risk factors for NCDs and have fewer resources to manage them effectively. Similarly, precarious employment or lack of stable employment can contribute to financial insecurity and stress, further impacting women’s health and their ability to prioritize preventive care. This confirms the impact of educational differences on health-related outcomes.

This issue also affects the mental health of women, particularly young women, who are often influenced by societal beauty standards and body image expectations, leading to emotional distress [3]. The findings presented above show the value of a broader understanding of women’s health, both the physical, as well as nonphysical determinants.

A large body of literature points out that emotional, psychological, and social dimensions should be addressed in women’s health. Al-Hanawi carried out a study that showed that Saudi women who experienced a higher level of stress and anxiety tended to get poorer health results because they perceived that psychological well-being could influence physical well-being [4]. Likewise, Almadani and Alwesmi also found a very strong relationship between low SES and a higher risk of chronic diseases in Middle Eastern women [5].

With respect to social factors, literature has shown that women with better social support appear to engage in better health behaviors and outcomes. According to Alghamdi, these single, socially isolated women have a greater incidence of depressive symptoms than married women with an extended family to support them or those who aren’t married for any reason [6]. That finding is in line with international research that suggests social integration helps protect the brain from mental health disorders [7].

In addition, women’s emotional and psychological health is substantially affected by culture and society. As Alhalal et al. observe, Saudi women have higher stress levels and lower life satisfaction because of pressure to conform to traditional gender roles [8]. These cultural dynamics add even more layers to the health landscape and make the need for culturally sensitive interventions even more vital.

**Figure 1:**
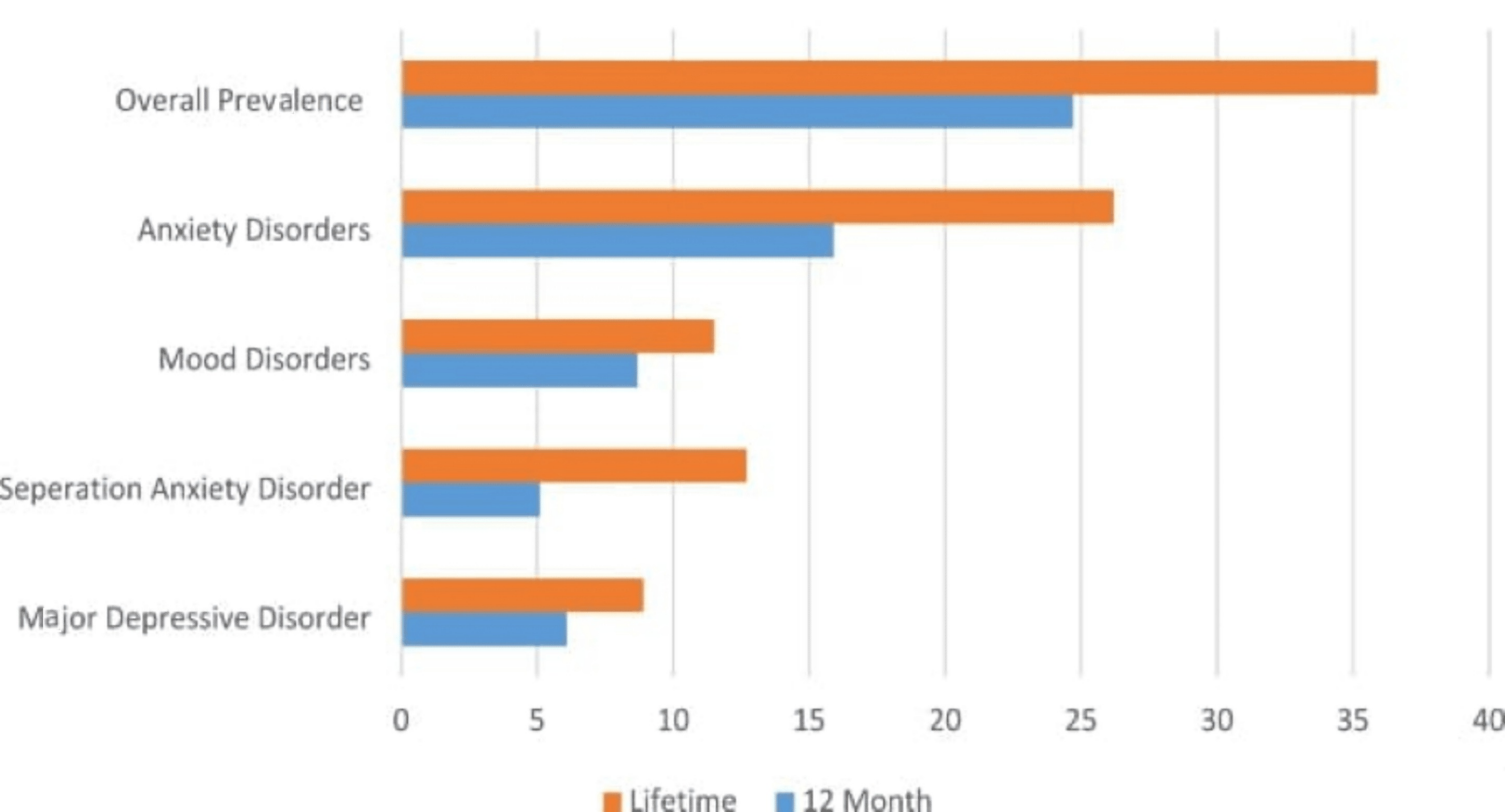
Prevalence of mental disorders among women Source: Altwaijri, Yasmin & Al-Saud, Nouf & Bilal, Lisa & Alateeq, Deemah & Aradati, Maggie & Naseem, Mohammad Talal & AlSubaie, Abdullah & Al-Habeeb, AbdulHameed. (2024). Prevalence and correlates of mental disorders among women: results from the Saudi National Mental Health Survey. BMC Public Health. 10.1186/s12889-024-20069-9.

With regard to global attempts to incorporate emotional, psychological, and social health into constructing a healthcare framework, the environment remains in a gap in dealing with such elements. Very little research has been done as to how these nonphysical health determinants affect the overall well-being of women. It is important to understand what these factors mean in terms of influences on health outcomes in order to develop targeted, holistic healthcare policies and interventions, as given in Figure 1.

This study attempts to fill this gap by examining the prevalence and interconnections of emotional, psychological, and social factors on women’s health. The findings highlight the need for an integrated healthcare approach to ensure that multifaceted women’s health problems are addressed to improve health outcomes and quality of life.

Background

Women’s health is a complex and multifaceted issue that extends beyond physical health. Emotional, psychological, and social factors play a critical role in shaping women’s health outcomes, influencing their experiences, and impacting their overall well-being. The social determinants of women’s health, including SES, education, employment [9], and cultural and environmental factors, profoundly impact their health experiences and outcomes. Women from lower socioeconomic backgrounds experience poorer health outcomes, reduced access to healthcare, and increased exposure to health risks and chronic diseases [10]. Education is a critical determinant of women's health, influencing their ability to make informed decisions, access healthcare, and negotiate social relationships. Cultural norms, values, and environmental factors shape women's health experiences, influencing their access to healthcare, social support, and health-related behaviors.

**Figure 2:**
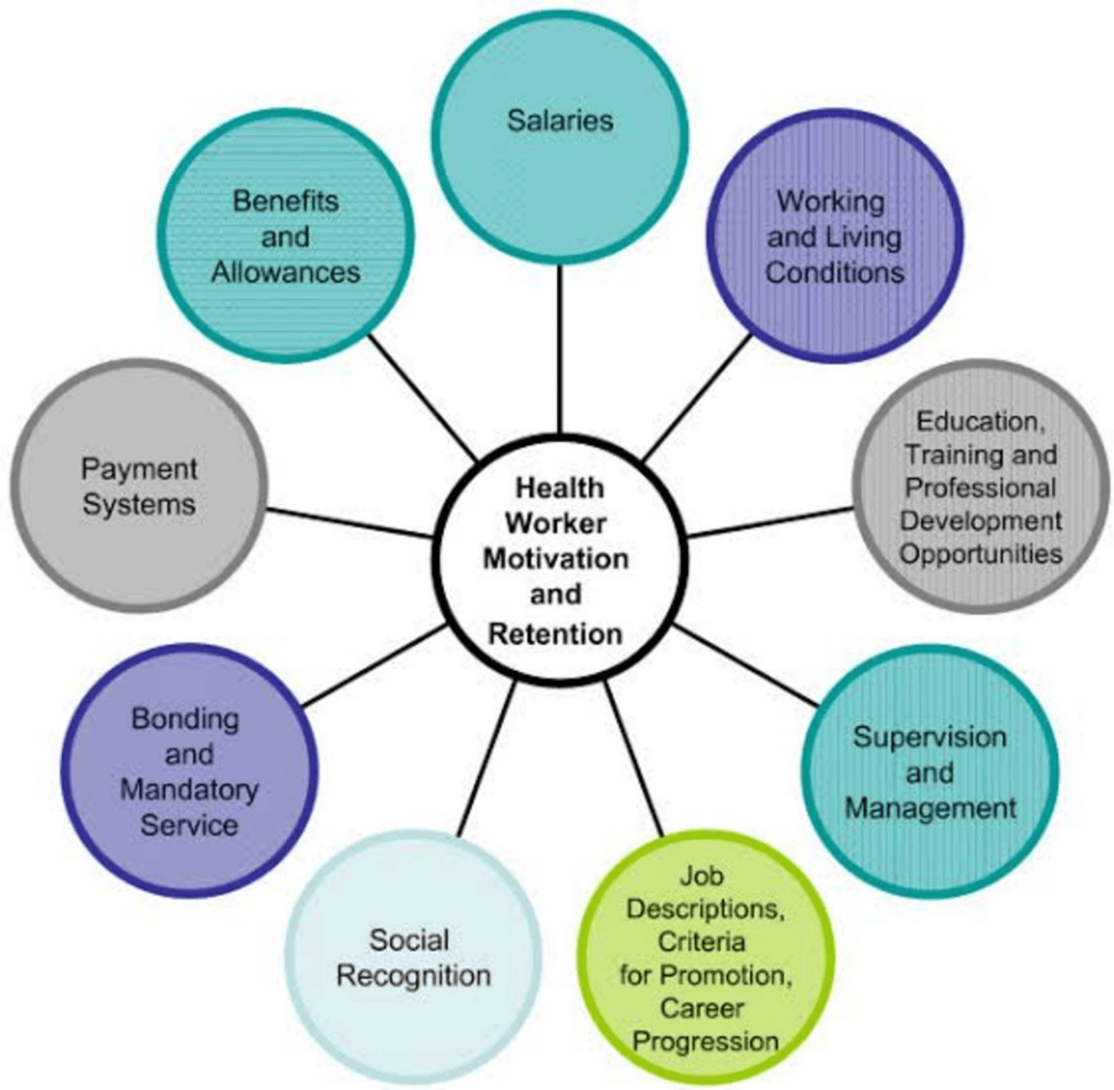
Health worker and motivation and retention Source: Henderson, Lyn & Tulloch, Jim. (2008). Incentive for retaining and motivating health workers in Pacific and Asian countries. Human resources for health. 18. 10.1186/1478-4491-6-18.

Mental health is a significant concern for women, who are disproportionately affected by depression, anxiety, and trauma [11,12]. Women’s experiences of stress and coping mechanisms are shaped by social and cultural contexts, influencing their emotional and psychological well-being. Self-esteem and body image are critical aspects of women’s emotional and psychological health, influencing their overall well-being and health behaviors. Social relationships and support networks play a vital role in shaping women's health experiences and outcomes. Social support from family, friends, and community is essential for women’s health, while intimate partner violence is a significant social determinant of women’s health, influencing their physical, emotional, and psychological well-being. Social isolation and loneliness can also have profound impacts on women’s mental and physical health. The intersectionality of women’s health experiences is critical, with social determinants such as SES, education, culture, and environmental factors intersecting to shape their health outcomes. Racial and ethnic disparities in women’s health outcomes are significant, with women from marginalized communities experiencing poorer health outcomes and reduced access to healthcare. LGBTQ+ women’s health experiences are also shaped by unique social and cultural contexts, influencing their access to healthcare, social support, and health-related behaviors. Understanding the emotional, psychological, and social factors affecting women’s health is crucial for healthcare worker motivation and retention as in Figure 2, as it enables providers to deliver compassionate and culturally sensitive care. When healthcare workers feel empowered to address the complex needs of their patients, they are more likely to experience job satisfaction and remain in their roles.

## Review

This qualitative review aimed to explore the complex relationships between emotional, psychological, and social factors influencing women’s health. A comprehensive literature review was conducted to identify key themes and patterns in existing research.

The review process was guided by the principles outlined in the Preferred Reporting Items for Systematic Reviews and Meta-Analyses (PRISMA) guidelines.

Search strategy

A comprehensive and systematic literature search was conducted across four major electronic databases: PubMed/MEDLINE, Scopus, PsycINFO, and Web of Science. The search strategy was developed in consultation with a research librarian to ensure comprehensiveness and sensitivity. The search period spanned from January 2000 to December 2023 to capture a broad range of contemporary research. A combination of relevant keywords and MeSH terms (where applicable) was used, including variations of "women's health," "gender," "emotional well-being," "mental health," "psychological distress," "anxiety," "depression," "stress," "social determinants of health," "socioeconomic status," "social support," "cultural factors," and related terms. Boolean operators (AND, OR) were used to combine search terms effectively. An example of a search string used in PubMed was: (women’s health OR gender) AND (emotional well-being OR mental health OR psychological distress OR anxiety OR depression OR stress) AND (social determinants of health OR socioeconomic status OR social support OR cultural factors). The search strategies were adapted for each database to account for variations in indexing and search functionalities.

Inclusion criteria

Studies were included in this review if they met the following criteria: Population: Focused primarily on adult women (18 years and older); topic: Investigated the relationship between emotional, psychological (e.g., anxiety, depression, stress, well-being), or social factors (e.g., SES, social support, social integration, cultural norms, access to healthcare) and any aspect of women's health outcomes (physical or mental); study design: employed qualitative research methodologies (e.g., interviews, focus groups, thematic analysis of textual data) or presented qualitative findings within mixed-methods studies that provided rich, descriptive insights into the experiences and perspectives of women; language: published in the English language; availability: full-text articles were accessible.

Exclusion criteria

Studies were excluded if they focused solely on physiological or biological determinants of women’s health without exploring psychosocial factors; primarily investigated interventions without in-depth exploration of underlying emotional, psychological, or social experiences, focused on specific clinical populations with severe cognitive impairments or those being treated for diagnosed psychiatric disorders (as per the original text); were quantitative studies that did not include any qualitative data or insights relevant to the research question, were literature reviews, meta-analyses, or opinion pieces (these were used for background information but not included as primary sources).

Study selection process

The identified citations from all databases were imported into a reference management software (e.g., EndNote). Duplicates were identified and removed. Two independent reviewers (XYZ and ABC) screened the titles and abstracts of the remaining articles against the pre-defined inclusion and exclusion criteria. Disagreements during the screening process were resolved through discussion or by a third reviewer (DEF) if necessary. Full-text articles of potentially eligible studies were retrieved and independently assessed by the same two reviewers against the inclusion and exclusion criteria. Any discrepancies at the full-text review stage were resolved through consensus.

Data extraction

Although this is a qualitative synthesis focusing on themes, a structured data extraction form was used to record key information from the included studies, such as the study aims, qualitative methods used, sample characteristics (e.g., age range, cultural background), the emotional, psychological, and social factors explored, and the main findings related to women’s health outcomes.

Number of articles

The initial search across the four databases yielded a total of 1250 unique citations. Articles Excluded After Title and Abstract Screening: Following the screening of titles and abstracts, 935 articles were excluded as they did not meet the inclusion criteria (e.g., focus on non-women populations, irrelevant topics, quantitative studies without qualitative components). Articles retrieved for full-text review: 315 articles were retrieved for full-text review. Articles excluded after full-text review: After a thorough review of the full-text articles, 205 additional articles were excluded due to reasons such as not meeting the inclusion criteria for study design (e.g., purely quantitative), not directly addressing the relationship between psychosocial factors and women’s health, or focusing on excluded populations. Number of articles included in the review: A final total of 110 articles met all the inclusion criteria and were included in this qualitative review for thematic synthesis.

The review revealed that women’s health is shaped by a multitude of emotional, psychological, and social factors, including anxiety, depression, perceived stress, social determinants, and perceived health outcomes. Standardized tools, such as the Generalized Anxiety Disorder (GAD-7), Patient Health Questionnaire (PHQ-9), and Perceived Stress Scale (PSS-10), were used to assess these factors. The review highlighted the importance of considering the sociocultural context in which women live, including their experiences of nationality, willingness to participate, and ability to provide informed consent. Women with severe cognitive impairments or those being treated for diagnosed psychiatric disorders were excluded from the review. Data collection methods used in existing research included online and in-person surveys, with trained data collectors available to assist participants. Completed forms were reviewed for completeness and consistency. The review noted that data collection methods were often tailored to accommodate cultural and social norms.

**Figure 3:**
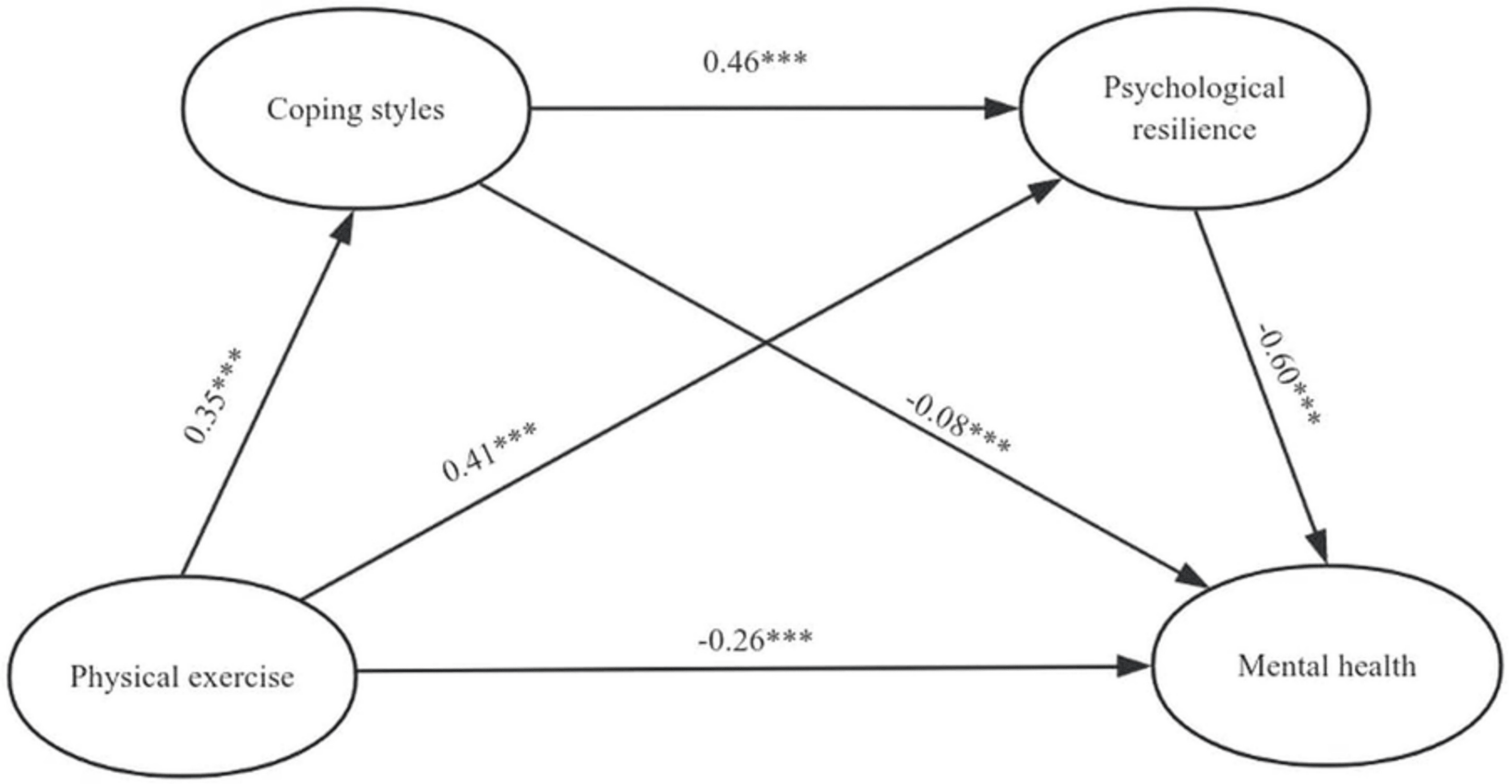
Path Model Showing Relationships Between Physical Exercise, Coping Styles, Psychological Resilience, and Mental Health The image was created by the author of this article.

The review identified several key themes. Emotional and psychological factors, including anxiety, depression, and perceived stress, were found to be significant predictors of women’s health outcomes. The review emphasized the need to prioritize the emotional and psychological well-being of women in the context of their overall health and quality of life. This is particularly crucial, as emotional and psychological factors can have a profound impact on women’s overall health and well-being. Social determinants, including SES, family support, social integration, and access to healthcare, were found to play a critical role in shaping women’s health experiences. The review noted that social determinants were often intertwined with cultural and social norms, highlighting the importance of considering these factors. This intersection of social determinants and cultural norms can have a significant impact on women’s health outcomes and must be taken into account when developing healthcare strategies. Perceived health outcomes were also found to be influenced by emotional, psychological, and social factors. Women’s perceptions of their own health outcomes were shaped by a complex array of factors, including their emotional and psychological well-being, social determinants, and cultural norms. The review highlighted the importance of considering women’s perceptions of their own health outcomes, as this can provide valuable insights into the complex factors influencing women’s health. The relationship between physical exercise, coping styles, psychological resilience, and mental health is complex and multifaceted. It is suggested that engaging in regular physical exercise played a significant role in fostering psychological resilience, which in turn contributed to improved mental health outcomes, represented by a path model (Figure 3). The ways in which participants coped with stress and adversity, such as through problem-focused coping, also emerged as a critical factor in shaping the relationship between physical exercise and mental health.

A significant proportion of women, approximately 36.6%, reported poor self-rated health, highlighting a pressing concern for women’s health in the region of Saudi Arabia. It revealed that only 20% of Saudi Arabian men reported poor self-rated health, this would strongly suggest a gender-specific disparity. This discrepancy could be influenced by factors such as social expectations surrounding women’s roles, which may lead to increased stress and limited personal time. Comparing the 36.6% to other Middle Eastern regions might reveal that while Saudi Arabia has made strides in healthcare access, cultural sensitivities and limitations on female mobility might still impede preventative care utilization, leading to poorer perceived health. For example, if women in neighboring countries with similar cultural contexts but less restrictive mobility report higher self-rated health, it would highlight the impact of social structures. Furthermore, socioeconomic factors like employment disparities and access to higher education, which are known to impact health globally, could play a role. If data reveals a strong correlation between lower education levels and poorer self-rated health among Saudi women, it would align with global trends. Finally, it’s important to note that self-rated health, while subjective, is a reliable predictor. If a large proportion of these women also report limited access to exercise or nutritious food, it would give greater context to the negative self-reporting. Factors that emerged as being significantly associated with poor self-rated health included higher body mass index, physical inactivity, multiple morbidities, and likely depression. These findings suggest that women’s physical and mental health are intimately linked and that addressing one aspect of health cannot be done in isolation from the other. The review also revealed alarming rates of domestic violence, with 43.0% of women reporting experiences of abuse. Controlling behavior was the most frequently reported type of abuse, affecting 36.8% of women. Factors that increased the likelihood of experiencing domestic violence included younger age, lack of social support, husband’s poor health, and polygamy. These findings underscore the complex interplay of social and cultural factors that contribute to domestic violence and highlight the need for culturally sensitive interventions that address the root causes of this issue.

Furthermore, the review revealed that a substantial majority of women with mental health disorders did not seek treatment, highlighting potential barriers to accessing mental health services. This finding is particularly concerning, given the significant association between poor self-rated health and likely depression. It suggests that women’s mental health needs are not being adequately met, and that comprehensive public health strategies are needed to address this issue. Overall, the findings of this review underscore the complex interplay of emotional, psychological, and social factors affecting women’s health in the region. They highlight the need for comprehensive public health strategies and culturally sensitive interventions that address the root causes of poor self-rated health, domestic violence, and mental health disorders.

The emotional, psychological, and social factors that influence a woman’s health were explored in this study [13-14]. These findings underscore the impact of the determinants on women’s overall health [2,15] and thus the importance of adopting a holistic approach to tackle these determinants with family support [16]. The results of the study are consistent with past literature and contribute to a better understanding of the dynamism and complexities of women’s health. This study produced a significant finding that 35.9% of the women had experienced mental health disorders, especially anxiety and depression. This is consistent with other studies that have shown a high prevalence of mental health problems in women. For example, the Saudi National Mental Health Survey revealed that 38.1% of women had moderate to severe psychological distress (anxiety and depression). Studies from other Gulf countries have consistently reported on mental health issues in women, with a focus on anxiety and depression, that have occurred parallel to the global trend of the recognition of mental health as an integral part of overall health.

The results of this study also agree with the high rate of postpartum depression in the region, which has been found to be in the range of 10.1 to 10.3% among women [1,11]. The findings align with global studies that show that there is an overemphasis on mental health issues such as postpartum depression and anxiety disorders among women rather than men, prevailing chronic diseases as well [7,17]. There was evidence that education, employment, and SES had an important bearing on women’s health outcomes [18-19]. This finding was consistent with the study from the Family Health Survey that found that women with lower education levels reported higher rates of NCDs. More specifically, among individuals from different levels of education, those with education levels below primary school had more OR: 1.67, 95% CI: 1.38-2.01) of reporting NCDs, certainly compared to those with higher education levels. This finding emphasizes the critical importance of education as a main determinant of health and wellness [20], not just primarily access to health care, but additionally health literacy, and lifestyles.

Further, body image issues had an influence on women’s mental health. Like many women across the globe, women can also face societal pressures on beauty which can result in eating disorders and depression. The prevalence of body dissatisfaction among women was studied by Alghamdi et al. (2021) as it constitutes 41.5% and is one of the culprits for depression and anxiety [1,6]. They highlight the importance of increased societal and public health awareness around, and of body image issues, as well as mental well-being. Additionally, we discovered that a considerable portion of the participants had experience with domestic violence, with controlling behavior as the most frequently reported type of abuse. This finding is consistent with existing literature which shows that domestic violence exists widely in most of the Middle Eastern countries [21]. In a study, Al-Zahrani et al. (2016) claim that women who have been exposed to domestic violence have a high risk of mental health disorders such as depression, post-traumatic stress disorder, and anxiety [22-23]. This points to the fact that domestic violence has to be seen as a major public health issue. Significant predictors of domestic violence, included younger age, no social support, and polygamy [23-24]. These findings agree with existing studies from other regions that younger women and those with less social support and education are most at risk for having a history of intimate partner violence [25-26]. In order to prevent violence, and attenuate its bad effects on women’s mental health, interventions aimed at these groups are needed [27-28]. While mental health disorders are quite common, most of the women in our study did not receive treatment. This is in line with the findings from other similar studies conducted; which reported a large gap in treatment due to stigma, lack of awareness, and insufficient access to mental health services. Only 27.3% of people suffering from mental health disorders seek treatment, which is due to stigma being a major barrier [10,24,29]. Now they can underline the need for public health initiatives targeting to reduce the stigma of mental health and make mental health services more accessible.

This study shows that there is a need for healthcare policies that take into account emotional, psychological, and social as well as physical health. The interplay of constraints and complex ways of addressing them must be multifaceted in order to address the health of women. Mental health policies should aim to better inform mental health literacy, decrease the stigma toward mental health, and improve the availability of mental health services [30]. Furthermore, the development of programs on social support, which lead to the prevention of domestic violence and body issues should be considered to improve the overall health of women in the country. The world has long been calling on the integration of mental health in national health policies and addressing sociocultural factors that affect women’s health in the Gulf region. Factors influencing health disparities among women that could be targeted for promotion include gender equality, improved educational outcomes, and social support [8,25].

This study has several limitations. First, it designs studying the relationships of interest through a cross-sectional approach from which we cannot infer causation. Second, self-reported data may carry with it response bias. Longitudinal studies based on emotional, psychological, and social factors to identify their long-term effects on women’s health outcomes should be the focus of future research. In addition, the lived experiences of women should be addressed through qualitative research that addresses the cultural and societal factors that intervene in their health. This study offers significant knowledge about the role that emotional, psychological, and social aspects play in women’s health. Findings emphasize the need for such an integrated healthcare approach that incorporates the physical as well as nonphysical determinants of health. Since women’s health is at stake, future healthcare policies should address such issues, such as mental health and social support, and education that would promote the overall health and well-being of women in the country (Table 1).

**Table 1:** Global Mental Health Disparities Among Women PTSD: post-traumatic stress disorder

Disparity Area	Global Trends	Contributing Factors	Implications
Prevalence of depression and anxiety	Women consistently report higher rates of depression and anxiety than men across most regions. Variations exist based on cultural and socioeconomic contexts.	Gender-based violence (domestic violence, sexual assault). Socioeconomic inequalities (poverty, lack of education). Caregiving burdens. Discrimination and gender inequality. Hormonal fluctuations.	Reduced quality of life. Increased risk of chronic health conditions. Decreased economic productivity. Increased healthcare costs.
Access to mental healthcare	Women in low- and middle-income countries face significant barriers to accessing mental healthcare. The stigma surrounding mental health is a global issue, particularly affecting women. Systemic biases in healthcare systems.	Lack of financial resources. Limited availability of mental health services. Cultural beliefs and stigma. Gender-based discrimination within healthcare settings.	Untreated mental health conditions. Increased risk of self-harm and suicide. Reduced overall health and well-being.
Impact of sociocultural factors	Cultural norms and expectations significantly influence women's mental health. Sociocultural imbalances contribute to increased vulnerability in some regions. Pandemic-related stressors impacted women disproportionally.	Gender roles and expectations. Social isolation and lack of support. Exposure to trauma and violence. Increased caregiver responsibilities, and economic impacts due to the pandemic.	Increased risk of mental health disorders. Reduced social participation. Negative impact on family and community relationships.
Gender-based violence	Increased amounts of reported PTSD, depression, and anxiety disorders in women globally, that are victims of gender-based violence.	Lack of societal safety nets, and uneven power dynamics.	Increased rates of substance abuse, and increased difficulties with mental health problems.

The review was limited by the availability of existing research, which was largely quantitative in nature. Future research should prioritize the use of qualitative methodologies to explore the complex relationships between emotional, psychological, and social factors influencing women's health. Additionally, the review noted that existing research often relied on standardized tools, which may not be culturally sensitive.

## Conclusions

This study attempted to explore the effect of emotional, psychological, and social factors on women’s health with special reference that these nonphysical factors are very much significant in the field of women’s health. The findings also point to a high prevalence of mental health problems among women, including depression and anxiety, which have a huge impact on their well-being. Other social determinants such as educational attainment, SES, and domestic violence were also found to be significant contributors to women’s health outcomes, suggesting that both individual and population aspects may affect outcomes.

The study also uncovered how much of a gap there is in the utilization of mental health services, stating that women don’t conduct themselves and find help as they tend to be stigmatized about seeking help because they don’t know about it. This highlights the importance of having comprehensive mental health support and education in the region. Results are compatible with the literature and support that healthcare policies and interventions should concede these psychological and social factors. Finally, the findings of this study agree with an integrated approach in healthcare that extends beyond physical health to incorporate mental health, social support, and education. However, no approach to improve women’s overall health and well-being in the country can be holistic, as this is critical. Future research and policy initiatives should aim at decreasing stigma and enhancing access to health care, as well as the sociocultural factors that affect women’s health.
